# To lie or not to lie: Super-relaxing with myosins

**DOI:** 10.7554/eLife.63703

**Published:** 2021-02-10

**Authors:** Suman Nag, Darshan V Trivedi

**Affiliations:** 1 Department of Biology, MyoKardia IncBrisbaneUnited States; 2 Department of Biochemistry, Stanford University School of MedicineStanfordUnited States; Stanford University School of MedicineUnited States; Stanford University School of MedicineUnited States

**Keywords:** Myosin, Super-relaxed state, Interacting heads motif, Hypertrophic cardiomyopathy, Mavacamten, Muscle Contraction

## Abstract

Since the discovery of muscle in the 19th century, myosins as molecular motors have been extensively studied. However, in the last decade, a new functional super-relaxed (SRX) state of myosin has been discovered, which has a 10-fold slower ATP turnover rate than the already-known non-actin-bound, disordered relaxed (DRX) state. These two states are in dynamic equilibrium under resting muscle conditions and are thought to be significant contributors to adaptive thermogenesis in skeletal muscle and can act as a reserve pool that may be recruited when there is a sustained demand for increased cardiac muscle power. This report provides an evolutionary perspective of how striated muscle contraction is regulated by modulating this myosin DRX↔SRX state equilibrium. We further discuss this equilibrium with respect to different physiological and pathophysiological perturbations, including insults causing hypertrophic cardiomyopathy, and small-molecule effectors that modulate muscle contractility in diseased pathology.

## Introduction

Enzymes, an epitome of exquisite evolutionary design, can modulate their activity by intricate conformational changes induced by upstream events that reflect the dynamic requirements of a cell. There are tens of thousands of such intricate, well-designed machines that are dynamically switching between the active and the inactive state to regulate their function. Many pharmaceutics, for example, those that target the G-protein-coupled receptors, takes advantage of such regulation. Amongst this vast universe of enzymes, the myosin family of mechanoenzymes, which are classified as molecular motors, performs mechanical work by an ATP-mediated cyclic interaction with actin and are responsible for muscle movement, cell division, intracellular cargo transport, passive force generation, and many more cellular functions (Hartman and Spudich, 2012). In the cardiac and skeletal muscles, myosins form a part of the thick filament, which slides across actin-containing thin filaments, thereby producing the necessary force required for muscle contractility. This contracting and relaxing apparatus, called the sarcomere, is primarily believed to be regulated by calcium, where the troponin complex on the thin filament serves as the calcium sensor (reviewed by Kobayashi and Solaro, 2005). The binding of calcium to this sensor triggers a series of conformational changes in the troponin–tropomyosin system of the thin filaments and opens up myosin-binding sites on actin. This results in actin–myosin interaction that leads to muscle contraction. Conversely, upon calcium efflux from the muscle cells, the troponin–tropomyosin system of the thin filaments blocks the actin–myosin interaction, thereby causing muscle relaxation. This regulation is even strongly realized in the cardiac muscle on a beat-to-beat basis. In addition to this well-designed evolutionary mechanism, recently, the thick-filament-mediated regulation of the sarcomere has also been discovered and is the focus of much attention. Like other enzymes in biology, the thick filament of the sarcomere is now thought to have at least an active and a dormant conformation of myosin. A dynamic equilibrium between these conformational states is believed to be essential to modulate the sarcomere contraction. Mutations affecting this equilibrium have been found to cause hypercontractility of the cardiac sarcomere. Importantly, myosin-binding small-molecule effectors have been demonstrated to reverse this equilibrium and revert the hypercontractile phenotype of the sarcomere. Other than myosins of the sarcomere, unconventional myosins, such as myosin V, also modulate their activity by transitioning into a dormant state (Liu et al., 2006). Thus, the structure–function relationship plays a foundational role in modulating such enzymatic activity and related downstream processes.

### The super-relaxed state of myosin – an evolutionary mechanism to regulate muscle contraction

**Figure 1.**
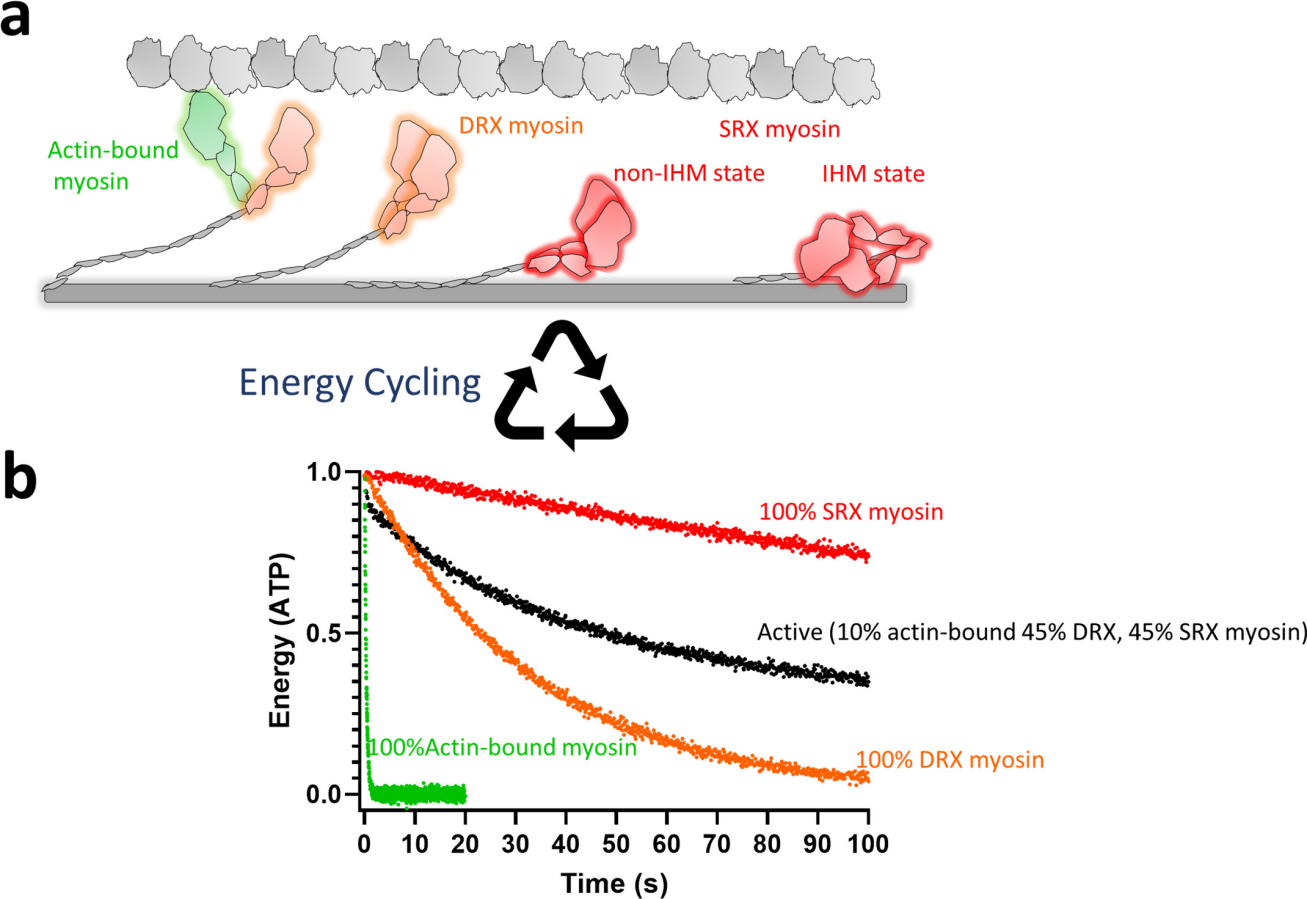
Schematic representation of the possible different functional states of myosin. (**a**) Highlighted in green are actin-bound myosins, which are most active and utilize maximum ATP. This myosin state is in equilibrium with an off-actin state, shown in orange, the DRX state of myosin, which has 100-fold less activity than the actin-interacting form. Myosins in the DRX state are in equilibrium with those in the SRX state, as shown in red, which has a further 10-fold less activity than the DRX state. The SRX state of myosin could be due to either a traditional IHM state or a non-IHM state. Actin is shown as gray globular domains, and the myosin thick-filament shaft is shown as a gray cylindrical rod. (**b**) Simulated energy (ATP) utilization by different myosin states is shown. The ATP turnover rate for the actin-bound myosin, DRX myosin, and SRX myosin was assumed to be 3 s^−1^, 0.03 s^−1^, and 0.003 s^−1^, respectively. A standard deviation of 0.1 units was used to generate random Gaussian noise. The simulated curves were generated using the GraphPad Prism software.

Ten years ago, a seminal breakthrough was made when Roger Cooke and colleagues discovered a new functional state of myosin molecules in rabbit skeletal muscle fibers, which they termed the super-relaxed(SRX) state (Stewart et al., 2010). Multiple subsequent studies have confirmed this SRX state in cardiac and skeletal fibers from many different species (Hooijman et al., 2011; McNamara et al., 2015; Naber et al., 2011; Anderson et al., 2018). The population of the SRX state is measured kinetically by following the release of fluorescent ATP hydrolysis products from myosin. A hallmark of this SRX state is an ultra-slow release of the ATP hydrolysis products relative to myosins in other states. In other words, in the muscle sarcomere, myosin is thought to exist in three different functional states (Figure 1). The first is the active cycling state of myosin, which involves actin-activated ATP hydrolysis and a rapid ATP turnover time of <1 s. The second state, often called the disordered relaxed (DRX) state , is the ATP turnover state of myosin in the absence of actin, which has a very slow ATP turnover time of <30 s. The third, or the SRX state, is the state that has even more prolonged ATP turnover times of more than 100 s. As an analogy, myosin in the actin-bound active state would be equivalent to a car racing on the freeway, the DRX state would be equivalent to a car idling at a stoplight, and an SRX state would be a car parked with its engine off.

During muscle contraction, myosins are continuously shuttling between the actin-bound state and the actin-free DRX state (Figure 1). In contrast, the myosins in the SRX state are held ‘in-reserve’. In skeletal and cardiac muscle fibers, it has been estimated that 50–60% of the myosin heads are in the SRX state and are hypothesized to not participate in contractility (Stewart et al., 2010; Hooijman et al., 2011). However, one pertinent question that remains to be answered is whether these SRX myosins are pulled into action under the dynamic needs of the muscle (Brunello et al., 2020). Another outstanding question is, what is the structural basis of the SRX state? Although there is no atomic-level structure of myosin in the SRX state, researchers have previously discovered a folded-back structural state of myosin, which is termed the interacting-heads motif (IHM), where the two myosin subfragment 1 (S1) heads of myosin asymmetrically interact with one another and fold back onto its subfragment 2 (S2) tail (Woodhead et al., 2005; Zoghbi et al., 2008; Wendt et al., 1999; Wendt et al., 2001; Burgess et al., 2007; Jung et al., 2008a; Jung et al., 2008b) (reviewed in Trivedi et al., 2018 and Alamo et al., 2018). This state was discovered using cryo- and conventional electron microscopy in intact thick filaments isolated from various types of muscle and purified myosins in solution. For example, the early concept of regulating myosin contractility by forming the folded-back conformation was discovered in non-muscle (Craig et al., 1983) and smooth muscle myosin (Trybus et al., 1982; Onishi and Wakabayashi, 1982). In fact, the first 3D structure of this folded-back conformation was reported in smooth muscle myosin by Wendt et al., 1999. Many researchers have put forward an appealing hypothesis that the SRX state is related to this IHM state (Hooijman et al., 2011; Alamo et al., 2016; Cooke, 2011; Nogara et al., 2016a; Wilson et al., 2014) in which the folded-back myosin heads lie plastered to the shaft of the thick filament and have an ultra-low turnover of ATP (Figure 1). These heads are structurally away from actin and are kept in reserve. This IHM-like state has been evolutionarily conserved as a myosin-II inhibition mechanism and has been reviewed elsewhere (Trivedi et al., 2018; Alamo et al., 2018; Lee et al., 2018). However, many recent biochemical studies have shown that there are myosin states other than IHM that are SRX states (discussed below) (Figure 1). Along that line, the exact relationship between the SRX and IHM states remains an active area of investigation.

How does nature make use of this myosin IHM state to its advantage? It has been hypothesized that physiological response, such as myosin regulatory light chain (RLC) phosphorylation or myosin-binding protein-C (MyBPC) interaction (Trivedi et al., 2018) or any pathophysiological state, such as hypertrophic cardiomyopathy (HCM) that results in higher contractility, may involve recruitment of sequestered IHM heads into play. However, as discussed above, no studies directly demonstrate a structural corollary of the SRX and IHM states, although it seems likely that the structural IHM state is a possible source of the functional SRX state in the muscle. In the absence of direct structural information, many studies have focused on understanding the DRX↔SRX state equilibrium. Below we review several physiological, pathophysiological, and non-physiological factors known to modulate the SRX state of myosin and provide an evolutionary perspective of the importance of the myosin DRX↔SRX state equilibrium in both healthy and diseased conditions.

## Muscle physiology and the myosin SRX state

### Myosin RLC regulates the myosin SRX state

It has long been known that phosphorylation–dephosphorylation of RLC acts as a binary ‘on’–‘off’ switch to activate smooth muscle myosin activity (reviewed in Trivedi et al., 2018). There is a strong propensity of unphosphorylated smooth muscle myosin to fold to the IHM state. Striated muscle myosins are not switched ‘on’ and ‘off’ in this fashion. However, RLC phosphorylation is known to fine-tune striated myosin function in several different ways (Yu et al., 2016). Early studies have shown that phosphorylation affects the arrangement of myosin heads in skeletal muscle filaments (Levine et al., 1996). There is also evidence of dysregulation of RLC phosphorylation in different heart failure models and dilated cardiomyopathy hearts (Huang et al., 2008; van der Velden et al., 2003; Toepfer et al., 2013). At the molecular level, multiple findings have led to the hypothesis that in the relaxed state of the striated muscle sarcomere, when a sub-population of RLCs are not phosphorylated, a fraction of myosin heads are held down on the filament backbone in which the myosin is considered to be in the IHM state (reviewed by Craig and Woodhead, 2006 and Alamo et al., 2018). Phosphorylation of the RLCs then causes these myosins to become more loosely associated with the filament backbone facilitating cross-bridge interactions with actin (reviewed in McNamara et al., 2015 and Trivedi et al., 2018), suggesting fine-tuning the functional ‘on’ and ‘off’ state of myosin. What does this mean in terms of the myosin DRX↔SRX state equilibrium? The first study to report the ability of RLC phosphorylation to depopulate the myosin SRX state was in rabbit skeletal muscle fibers (Stewart et al., 2010) and subsequently reproduced in tarantula skeletal muscle (Naber et al., 2011). A similar observation was made where a phosphomimetic version of the RLC, S15D, in HCM-causing R58Q RLC mutant background, depopulated the SRX state in skinned porcine papillary muscle fibers (Yadav et al., 2019). This report is discussed further under section ‘Diseased state and therapeutic modulators of myosin SRX state ’. In reconstituted full-length porcine cardiac myosin thick filaments, it has also been recently reported that either phosphorylation of the RLC with MLCK or depletion of RLC decreases the myosin population in the SRX state (Gollapudi et al., 2020a). All of these observations together suggest an RLC phosphorylation-mediated shift in DRX↔SRX state equilibrium toward the DRX state of myosin. This shift towards the DRX state has been correlated with the depopulation of the structural IHM state upon RLC phosphorylation (Alamo et al., 2016). The observation that factors that affect the RLC also affect the population of the SRX state is not surprising if one assumes that the IHM state is a major source of the SRX state, as the two RLCs bind to each other forming one of the major interfaces stabilizing the IHM state (Alamo et al., 2016).

### Myosin essential light chain may be essential for the myosin SRX state

The role of essential light chain (ELC) and how Ca^2+^ binding to ELC (in the scallop regulatory domain containing two light chains together with a short fragment of the heavy chain) regulates molluscan muscle contraction has been the focus of study in the laboratories of Andrew Szent-Gyorgyi and others (Szent-Györgyi, 1975; Fromherz and Szent-Györgyi, 1995; Chantler, 2006; Trybus, 1994). Ca^2+^ binding to ELC has been shown to enzymatically and structurally turn on the myosin from its IHM-like ‘off’ state (Woodhead et al., 2013). However, the role of ELC in cardiac, skeletal, or smooth muscle is not well understood. Several laboratories have demonstrated that a specific N-terminal extension of the ELC may play an essential role in regulating myosin motor function and force production in striated muscle (Kazmierczak et al., 2009; Muthu et al., 2011; Guhathakurta et al., 2015). Recently, the hypothesis that this N-terminal domain of the cardiac ELC works as a molecular linker switch of the actin–myosin interaction by regulating the DRX↔SRX state equilibrium was tested in the papillary muscle of mice engineered to express an ELC lacking its N-terminal 43 amino acids (Tg-Δ43). The lack of the N-terminus of the ELC in mice resulted in a significant increase in the population of myosin in the SRX state, hence decreasing the proportion of myosin heads that are available to interact with actin (Sitbon et al., 2020). In agreement, myosin purified from Tg-Δ43 hearts showed significantly decreased ATP utilization and low actin-activated myosin ATPase compared to that from wild-type (WT) hearts. Although the studies are limited, this observation postulates the role of ELC in regulating the myosin SRX state and demands more studies in the future to solidify the hypothesis. Altogether, both the light chains associated with striated myosins seem to play a defining role in controlling the population of myosin in the SRX state, thereby functioning as regulatory switches of muscle contractility.

### Age, sex, and hormonal regulation of the myosin SRX state 

In relaxed skeletal muscle, it has been estimated that ~50% of the myosins remain in the SRX state, and the remainder is in the DRX state (Stewart et al., 2010; Cooke, 2011). It is also known that independent of muscle size or the presence of neurological or muscular diseases, there is a progressive loss of muscle strength with aging, termed dynapenia. Such age-related impairment of skeletal muscle contractility is more prominent in post-menopausal women than in men of the same age group. Therefore, it is essential to understand if aging and sex can shift the myosin DRX↔SRX state equilibrium or change the properties of the SRX state. This hypothesis was tested in the psoas muscle of young (3–4 months old) and aged (26–28 months old) C57BL/6 male and female mice, and no significant effects on the SRX population as a function of age or sex (Phung et al., 2018) were observed. However, only for female mice, the ATPase cycling time of myosin in both the DRX and SRX states was significantly faster with age. It is known that the ovarian hormone estradiol is the key hormonal signal to skeletal muscle in females, and its deficiency is detrimental to myosin and muscle function. In an earlier study (Colson et al., 2015) by the same laboratory, single turnover experiments in the ovariectomized C57BL/6 mice revealed two distinct populations of myosin in the SRX and DRX states in a similar proportion to the populations seen in the control mice. However, the ATP turnover rate of the SRX population was significantly increased compared to the non-operative controls. This effect was reverted to the WT control by a 60 days chronic treatment of the ovariectomy mice with estradiol, but not by treatment of the isolated psoas muscle with estradiol, suggesting that estradiol-mediated signaling reversibly regulates slow ATP turnover by myosin. Overall, the authors of these studies argue that the aging of females, which leads to a depletion of the hormone estradiol, shifts the muscle myosin more toward a DRX-like state with faster ATP cycling. The authors hypothesize that this could result in a buildup of weakly bound actomyosin complexes, possibly competing with the existing strongly bound ones, thus leading to slowed cross-bridge kinetics and increased weakness with age. An alternate possibility could be that the shift toward more DRX heads may be compensatory for the loss of muscle fibers rather than the cause of contractility loss. These studies may hint at the molecular differences in muscle traits across genders and age groups, and many more investigations in this direction would be required to prove either hypothesis.

### The role of calcium in regulating the myosin SRX state

Ca^2+^ plays a significant role in a variety of biological processes in the body. One such well-studied process is the activation of skeletal and cardiac muscle contraction mediated by direct binding of Ca^2+^ to the actin–tropomyosin–troponin regulated thin-filament complex (reviewed by Ebashi and Endo, 1968, and Gordon et al., 2000). As discussed earlier, Ca^2+^ binding to scallop myosin destabilizes the myosin from the IHM-like ‘off’ state, a mechanism that has not been evolutionarily propagated in the vertebrate system. Some early work by the laboratories of Richard Moss and others has shown that the Ca^2+^ sensitivity of the rate of force development in vertebrate skeletal muscle is mediated in part by the RLC subunit of the myosin cross-bridge (Metzger et al., 1989; Metzger and Moss, 1992; Metzger and Moss, 1990; Metzger and Moss, 1991). Other studies have proposed the importance of divalent cations such as Ca^2+^ and Mg^2+^ in the stability of the myosin SRX state (Nogara et al., 2016a). It is only recently that a study has reported that Ca^2+^ and not Mg^2+^ binding to reconstituted porcine cardiac myosin filaments depopulated the myosin SRX state, thereby proposing Ca^2+^-mediated activation of the thick filament as an additional regulation of the vertebrate muscle (Sa et al., 2019). Whether this mechanism holds in all kinds of skeletal and cardiac systems is debated (Irving, 2017) and remains an active area of investigation. If truly, Ca^2+^-mediated regulation of the myosin SRX population holds any ground, it would be yet another evolutionary way to regulate muscle contractility, especially in the context of the beating heart. More studies probing the role of Ca^2+^ in myosin regulation are needed.

### Muscle energetics and the role of ADP in governing the myosin SRX state

Phenotypically, based on properties such as contractile force and velocities, skeletal muscle fibers can be categorized as slow or fast, depending upon the different myosin isoforms that they express. Therefore, the whole-muscle metabolic rates and performance depend upon the distribution and proportion of these different muscle types. It has recently been shown that the fast skeletal muscle type has a lesser population of myosins in the SRX state than the slow muscle type (Phung et al., 2020), suggesting higher energy consumption by the fast muscle types. This higher resting energy consumption by the fast muscle fibers may contribute to differences in whole-body basal metabolic rate and provide additional insights for understanding muscle energetics.

Additionally, energy-related dysfunctions underlie many common diseases, such as mitochondrial disorders, which often impact the body’s top energy-consuming organs such as skeletal and cardiac muscle, brain, liver, and kidneys. For example, in the context of cardiac contractility, ATP-dependent enzymes are required for both systole (e.g., myosin) and diastole (e.g., SERCA). It has been guessed that ~60–70% of the ATP generated in the cardiac muscle is thought to be utilized by the myosins (Barclay, 2015). Therefore, any loss in chemical energy would lead to a failing heart that cannot meet the hemodynamic requirements of the body. It is important to note that the amount of ATP made and used per minute is many times greater than the size of the existing ATP pool, emphasizing that maintaining a high ATP supply is critically important for maintaining cardiac function. It is conceivable that hypercontractility of the heart either due to disease conditions such as HCM or effects of inotropes could disturb the [ADP]/[ATP] ratio and causes metabolic imbalances (Ingwall and Weiss, 2004). A couple of studies have reported the role of ADP in the manifestation of the myosin SRX state. For example, in rabbit fast and slow skeletal muscle fibers, ADP binding to the myosin attaches the myosin motor strongly to actin and displaces the regulatory proteins, activating the fiber. This action results in a possible strain-mediated depopulation of the SRX state of myosin and, thereby, attachment of more myosin heads to actin (Stewart et al., 2010). Similarly, upon ADP treatment, increased SRX state turnover rates were also observed in mouse psoas muscle fibers (Phung et al., 2018). These studies suggest that the thick filament in skeletal muscle, upon rigor-binding to actin, can undergo a strain-mediated destabilization of the SRX population. A similar mechanism was also observed in myosins of rat soleus slow skeletal myofibrils (Nelson et al., 2020). However, Hooijman et al., 2011 observed that such ADP-mediated destabilization of the SRX population in cardiac tissue is affected to a lesser extent by rigor-ADP states of adjacent myosin heads than is observed in skeletal fibers. The authors argue that this could be explained by a much weaker cooperative nature of the myosins in cardiac muscle as compared to those in the skeletal muscle. In addition, many more factors such as the differences in isoforms and phosphorylation levels of other proteins such as myosin RLC, MyBPC, and titin could contribute to these differences in the myosin cooperativity and is an open area of investigation.

Very interestingly, in skeletal muscle, when the ADP-mediated dysregulation of the myosin SRX state was studied at long sarcomere length (without actin–myosin overlap), ADP did not alter the number of myosins in the SRX population but significantly increased the ATP turnover rates of these myosins, suggesting that binding of ADP to some myosin heads increases the rate of release of phosphate from adjacent heads (Stewart et al., 2010). Recently, using porcine cardiac myosin, Gollapudi et al., 2020a have also shown an ADP-mediated destabilization of the SRX population of myosin in reconstituted myosin thick filaments, but not in the myosin subfragment S1 or heavy meromyosin (HMM), suggesting a relay mechanism of a cooperative destabilization of the SRX population, via the proximal and distal tails of myosin within the thick filament, which is absent in the shorter myosin constructs. These studies are consistent with the observation that the binding of ADP produces an open configuration of myosin and disrupts the helical ordering of the thick filament in an overstretched rabbit psoas muscle fiber (Xu et al., 2003). All these results together hint at the cooperative mechanism of the thick filament where one myosin bound to ADP (and therefore not likely to be in the SRX state) can structurally and functionally decrease the population of other myosins in the SRX state (those that have still not lost its phosphate), thereby possibly turning adjacent myosin heads to a more disordered ‘on’ state. These observations may suggest that in many metabolic muscle disorders, including in HCM, where there is an accumulation of ADP in the body, myosins would be more in the active DRX-like state, thereby consuming even more energy than usual, hence exacerbating the effect.

### Temperature: turning up the heat to stabilize the myosin SRX population

Temperature plays a vital role in dictating the dynamics of almost all biological processes in the body, and it is not surprising that temperature would affect the myosin DRX↔SRX state equilibrium. For example, in skeletal muscle, higher temperature induces a higher proportion of myosin heads into the SRX state and slows the ATP turnover time for these myosins (Stewart et al., 2010), possibly by populating myosin in the pre-power stroke ADP.Pi state. Many temperature-dependent studies monitoring the conformation of myosin have also substantiated the formation of more myosins in the structural ‘off’ state and stabilization of the ordered helical structure of the thick filament with an increase in temperature (Xu et al., 2003; Fusi et al., 2015; Caremani et al., 2019). This is not surprising. Due to the hydrophobic effect, most protein–protein interactions are stronger at higher temperatures. However, in a purified bovine cardiac myosin system, Rohde et al., 2018 observed that with increasing temperature, the fraction of myosin in the SRX state decreased in an S1 system and had no effect in the HMM system, opposite of what was observed by the previous study of Stewart et al., 2010 in skeletal muscle fibers. Together these observations suggest that the network of molecular interactions that govern the SRX state in a more complicated muscle fiber system are different from ones in a purified HMM or S1 system, which may give rise to different temperature dependencies in different systems. There is also a possibility of an inherent difference between the population of SRX states in skeletal and cardiac muscle systems (Hooijman et al., 2011). A study of the changes in myosin SRX population with changes in temperature using different muscle types from various cold-blooded and warm-blooded animals would shed more light on the molecular interactions that regulate the SRX state formation and may help us comprehend muscle dynamics in hibernating animals.

### The ancillary MyBPC and its role in stabilizing the myosin SRX population

MyBPC is a multi-domain thick-filament-associated protein in skeletal and cardiac muscle known to modulate cross-bridge kinetics via phosphorylation of serine residues in the so-called M-domain. The C-terminus of MyBPC binds to the thick filament, and the N-terminus can interact with both the myosin head and actin filament. Several studies reviewed in McNamara et al., 2015 and Trivedi et al., 2018 have suggested a role of MyBPC in stabilizing the myosin heads near the myosin thick filament in an IHM-like ‘off-state’, and phosphorylation of the M-domain serine residues shifts the equilibrium more toward a disordered ‘on-state’. How does this correlate with the myosin DRX↔SRX state equilibrium? It was first reported in a study with skinned cardiac muscle fibers that homozygous but not heterozygous MyBPC knockout mice have a significant decrease of the myosin population in the SRX state as compared to WT (McNamara et al., 2016). Consistent with this top-down observation, a bottom-up approach with purified human β-cardiac myosin recently demonstrated that the population of myosin in the SRX state increased in the presence of the C0-C7 MyBPC fragment (Sarkar et al., 2020). Both these studies suggest that MyBPC increases the population of the myosin in the SRX state. This work is further discussed under the ‘HCM-causing and other mutations in myosin dysregulate the myosin SRX state’ section.

In another mouse study (McNamara et al., 2019), it was further shown that in a triple phosphomimetic version of cardiac MyBPC (DDD; serine→aspartate changes at residues 273, 282, and 302 of the M-domain of MyBPC), the myosin SRX population significantly decreased compared to WT and triple phospho-ablated versions of MyBPC (AAA; serine→alanine changes at residues 273, 282, and 302). On further investigation of the site-specific phosphorylations, the authors showed that a phosphomimetic version of the ser 282 site (ADA; serine→aspartate change at residue 282, and serine→alanine changes at residues 273 and 302) was sufficient to shift the DRX↔SRX state equilibrium toward the DRX state of myosin. Interestingly, the reverse phosphomimetic version (DAD; serine→aspartate changes at residues 273 and 302, and serine→alanine change at residue 282 sites) did not affect the myosin DRX↔SRX state equilibrium. Like earlier studies, treatment of WT preparations with protein kinase A reduced the myosin in the SRX state. In contrast, there was no effect in the phospho-ablated version of MyBPC preparations, bolstering the hypothesis that phosphorylation of MyBPC plays a regulating role in turning the myosin from a functional ‘off’ to a more functional ‘on’ state.

Given the actuality that MyBPC localizes to the C-zone in the sarcomere, a recent report utilized single-molecule fluorescent imaging of rat soleus skeletal myofibrils under relaxed conditions to measure the kinetics of ATP hydrolysis in different regions of the sarcomere (Nelson et al., 2020). The authors spatially resolved the hydrolysis of individual fluorescently labeled ATP molecules by myosins in the C-zone, D-zone, and P-zone. The D- and P-zones are zones of the thick filament towards the Z- and M-lines, respectively, and are devoid of MyBPC. The myosin ATPase lifetimes within the C-zone were significantly longer than those of the flanking non-MyBPC containing regions. Also, within the C-zone, two different myosin populations corresponding to the DRX and SRX states were observed, while in the non-MyBPC containing regions, all myosins were predominantly in the DRX state, suggesting that the strongly inhibited SRX myosins exist predominantly within the C-zone of the skeletal sarcomere. All these observations together build up a story where the binding of MyBPC to myosins in the C-zone of the striated sarcomere populates the SRX state, and MyBPC phosphorylation shifts this equilibrium more towards the DRX state. This observation falls in line with the structural regulation of the muscle thick filaments by MyBPC (reviewed in McNamara et al., 2015 and Trivedi et al., 2018) and adds MyBPC as an essential contributor that has evolved in striated muscle to fine-tune myosin contractility indirectly.

### Myosin subfragment 2: the tail tells a tale about the myosin SRX state 

As described above, and as early as 2008, a folded-back structural state of myosin, termed the IHM, was discovered (reviewed in Trivedi et al., 2018 and Alamo et al., 2018), where the two S1 heads of myosin asymmetrically fold back onto the S2 tail. Furthermore, at a molecular level, the S1 head has been shown to interact with the proximal part of the S2 biochemically (Nag et al., 2017). Many other interactions, such as the S1–S1 and RLC–RLC of the two myosin heads, are proposed to stabilize the IHM state. However, from electron microscopy studies, it is apparent that the S1–S2 interactions are required to form the myosin structural ‘off’ state. Does this S2 subfragment of myosin also alter the myosin DRX↔SRX state equilibrium? This question was addressed recently in a report where two different versions of human β-cardiac HMM were made. One contained the first 25 heptads of the proximal part of the S2 subfragment (25-hep), and the other contained only the first two heptad repeats of the S2 subfragment (2-hep). In single turnover ATP experiments, the 25-hep HMM showed a significant stabilization of the myosin SRX population to ~60%. In contrast, the SRX population in the 2-hep HMM was ~20%, suggesting that the proximal S2 sub-fragment of myosin shifts the myosin DRX↔SRX state equilibrium towards the SRX state. Independently, a similar conclusion was also reached by Rohde et al., 2018 while investigating the SRX population in bovine cardiac S1 and HMM. The S2 subfragment of myosin has also been demonstrated to bind to the N-terminus of the MyBPC (Gruen and Gautel, 1999) (reviewed in Trivedi et al., 2018). In a different study, a high concentration of proximal S2 added to mouse ventricular cardiac muscle fibers significantly reduced the number of myosin heads in the SRX state (McNamara et al., 2019). This effect was also observed in the triple phospho-ablated version of MyBPC (AAA; serine→alanine changes at residues 273, 282, and 302 in the M-domain of MyBPC) (McNamara et al., 2019). These observations suggest that exogenous S2 can push the DRX↔SRX state equilibrium toward the DRX state of myosin, most likely through disruption of MyBPC–myosin interaction, further signifying the physiological role of myosin-subfragment 2 (myosin S2) to populate the SRX state. For a long time, in striated muscle biology, the myosin S2 fragment along with the tail domain were thought to provide structural stability and a passive anchoring role to myosin in the thick filament. However, these biochemical studies have started illuminating that a myosin domain such as the S2, which is far away from the enzymatic site of the protein, can regulate muscle contractility by controlling the population of myosins in the SRX state, thereby providing an additional evolutionary switch for muscle contractility.

## Diseased state and therapeutic modulators of myosin SRX state

### HCM-causing and other mutations in myosin dysregulate the myosin SRX state

Mutations in the MYH7 gene, which encodes the β-cardiac myosin heavy chain, are responsible for 20–30% of HCM cases (Ho et al., 2015). These mutations are clustered in specific hotspots on the β-cardiac myosin molecule. Homburger et al., 2016 demonstrated that these hotspots consist of a positively charged surface called the myosin mesa, the converter domain, and the proximal-S2 tail region of myosin. HCM is associated with increased energy demands in cardiac muscle due to excessive use of ATP. Thus, it was hypothesized that HCM-causing mutations could depopulate the energy- and ATP-conserving SRX state of the cardiac sarcomere. Anderson et al., 2018 utilized cardiac fibers from minipigs harboring the HCM-causing mutation R403Q and demonstrated a depopulation of the SRX state by this mutation. This has been recently verified by Gollapudi et al., 2020a in reconstituted myosin thick filament from the heterozygous R403Q porcine model, containing an equal mixture of WT and mutant myosin. Anderson et al., 2018 further demonstrated the SRX state depopulation in cardiac fibers from a human heart harboring another HCM-causing mutation, R663H. This was the first experimental report indicating the depopulation of the SRX state by HCM-causing myosin mutations, which was only theoretically hypothesized earlier (Alamo et al., 2017). Additionally, Anderson et al., 2018 demonstrated that the SRX state signal in fibers originates from myosin molecules. They measured the SRX population in recombinant, purified human β-cardiac myosin and its dependence on ionic strength for the first time. A follow-up study by Adhikari et al., 2019 demonstrated the depopulation of the SRX state in recombinant human β-cardiac myosin constructs harboring mutations in the myosin mesa (R249Q, H251N) and converter (D382Y, R719W) hotspots of HCM mutations in myosin. Sarkar et al., 2020 most recently reported depopulated SRX state in recombinant human β-cardiac myosin harboring the R403Q and R663H mutations, thus extending the Anderson et al., 2018 fiber work.

Interestingly, in an in vitro assay, binding of cMyBPC fragment C0C7 populated the myosin SRX state in WT recombinant human β-cardiac myosin. However, the HCM mutant R403Q myosin, which dysregulates the myosin SRX state, does not bind to C0C7, and hence its SRX state was not normalized. Interestingly, the R663H HCM-causing myosin, which does bind C0C7, also did not show any induction of the SRX state (Sarkar et al., 2020). This indicates that mutations in myosin that may or may not abrogate binding to MyBPC can still inhibit the ability of MyBPC to revert the depopulation of the SRX state. Reconfirming observations by Anderson et al., 2018 and Adhikari et al., 2019, most recently, Toepfer et al., 2020 measured the SRX state in mouse cardiac left ventricular tissue (predominantly MYH6 – α-cardiac myosin) harboring R403Q, V606M, and R719W mutations and demonstrated depopulation of the SRX state in these samples. In the same study, they introduced these mutations in human induced pluripotent stem cell -derived cardiomyocytes and observed a similar significant depopulation of the SRX state in this cell-based assay. Thus, all these studies have collectively demonstrated, from muscle fibers to cellular myofibrils to purified myosin, a depopulation of the SRX state in myosin carrying HCM-causing mutations. The effect of mutations in myosin that increase activity by inhibiting the SRX state explains the puzzling observation that HCM, which is an apparent gain of function, is more prevalent than DCM, an apparent loss of function. In general, gain-of-function mutations are rare. HCM reflects a loss of function of the SRX state.

As mentioned above, the RLC–RLC interface may play an essential role in modulating the DRX↔SRX state equilibrium. A study by Nogara et al., 2016a introduced cysteine point mutations at various locations in mouse skeletal RLC, exchanged these mutant RLCs into rabbit skeletal muscle fibers, and measured the SRX state. The idea behind designing these mutations was inspired by looking at the RLC–RLC interface in the folded-back model of tarantula skeletal myosin (PDB 3DTP). Sequence alignment of the tarantula exoskeletal muscle and vertebrate skeletal muscle demonstrated conserved amino acids clustered at the N-terminal lobe of the RLC, which is involved in the RLC–RLC interaction. They strategically placed fluorescent probes at these single cysteine sites on the very N-terminus of the RLC, at the interface of the RLC–RLC interaction, and the very C-terminus of the RLC. These fluorescent probes were placed to sense the DRX↔SRX state transitions of myosin. These mutant RLCs were then exchanged into skeletal muscle fibers, followed by SRX state measurements. The mutations by themselves had a minimal effect on the stability of the SRX population. Probes attached to cysteine residues in a region called the EF hand, which coordinates divalent cations like Mg^2+^ or Ca^2+^ on the RLC, showed the greatest depopulation of the SRX state. This hints at the importance of divalent cations in promoting the SRX state of myosin. This aspect has been discusssed in the ‘The role of calcium in regulating the myosin SRX state’ section. Probes attached to a site in the RLC–RLC interface also depopulated the SRX state, while probes on the C-terminal regions of the RLC had a minimal effect on the the SRX state. Mutations in RLC have also been reported to cause HCM. One such mutation, R58Q, is located near the Ca^2+^ binding site of RLC. A study has shown reduced phosphorylation of RLCs harboring the R58Q mutation in rapidly frozen ventricular heart samples from transgenic mice (Abraham et al., 2009). A follow-up study by Yadav et al., 2019 demonstrated that, contrary to the widely held hypothesis that HCM mutations shift myosin to the DRX state, the HCM-causing R58Q mutation populates the SRX state in reconstituted porcine fibers and transgenic mouse papillary muscle samples to an even greater extent than WT. A phosphomimetic mutation S15D, when introduced in the R58Q background, was demonstrated to rescue the R58Q-induced adverse effects in reconstituted porcine fibers. While the R58Q RLC led to an increased population of the SRX state, the S15D-R58Q RLC resulted in a depopulation of the SRX state, thus shifting the population of myosin molecules from the SRX to the DRX state. These mutations caused changes only in the SRX/DRX populations and not in their single turnover kinetics.

Similarly, another work from the Szczesna-Cordary lab looked at the effect of HCM-causing mutations in ELC on the SRX state of myosin (Sitbon et al., 2020). In this study, they studied the effect of the ELC mutation A57G in an ELC-humanized mouse model of HCM. SRX state was significantly depopulated in these mice, but there were no significant changes in the lifetimes of the SRX or DRX states. Thus, these observations underline the importance of light chains in modulating the SRX state of myosin. Altogether these investigations show that single-point mutations across the myosin molecule, either naturally occurring which causes HCM or those engineered in the laboratory, affect the DRX↔SRX state equilibrium, thereby emphasizing the evolutionary importance of residues in different domains of the myosin molecule that directly contribute to the energetic fine-tunings of the muscle.

### HCM-causing mutations in MyBPC also dysregulate the myosin SRX state 

The other major genetic player that causes HCM is MyBPC. Truncation or missense mutations in the cardiac MyBPC gene are responsible for ~30–40% of HCM cases (Ho et al., 2015). McNamara et al., 2016 made mouse models with either a heterozygous or homozygous knockout of cMyBPC. The heart weight to body weight ratio of the homozygous knockout mouse was significantly higher than the WT mouse. However, no such change was observed in the heterozygous knockout model. Upon performing SRX state assays with isolated cardiac fibers from these animals, the authors discovered a significant depopulation of the SRX state in the homozygous fibers. In contrast, the heterozygous fibers showed similar SRX state values compared to the WT. Another work by McNamara et al., 2017 looked at samples from human patients with eight different HCM-carrying MyBPC missense or truncation mutations. These samples were compared to non-failing donor heart samples and HCM samples that were negative for sarcomeric mutations. They discovered that compared to the non-failing hearts, the samples harboring missense or truncation MyBPC mutations had a significantly depopulated the SRX state combined with a decreased rate of ATP turnover by myosin in the SRX state. They did not see significant depopulation of the SRX state in HCM samples with sarcomere-negative mutations. Recently, Toepfer et al., 2019 demonstrated that a graded loss of cMyBPC results in a subsequent graded loss of the SRX state. They developed three mouse models for this work, one with a heterozygous truncating mutation of cMyBPC, one with a homozygous truncating mutation of cMyBPC, and a third having the potential of RNAi-mediated knockdown of cMyBPC. The heterozygous fibers had a 50% decrease in the SRX state compared to a 94% decrease in the homozygous fibers. It is worth noting that that truncating mutation in cMyBPC gives rise to haploinsufficiency, so that a 50% reduction in the SRX state maps to a 50% reduction in MyBPC protein expression. In this study, they also measured the SRX state in skinned human HCM heart fibers with heterozygous cMyBPC truncations. Once again, they observed a ~50% decrease in the SRX population of these mutant fibers compared to the WT human fibers. These findings that HCM-causing mutations in MyBPC dysregulate the myosin SRX state not only explains the cardiac hypercontractility in patients but also sheds light on the action of MyBPC, whose exact role in muscle contractility has been mysterious for many decades.

### Therapeutic approaches modulate the myosin DRX↔SRX state equilibrium

Decades of research on sarcomeric proteins have enhanced our understanding of the molecular underpinnings of force generation by the sarcomere. This has enabled small-molecule targeting of the sarcomere as a novel approach to modulate muscle contractility (Manstein and Preller, 2020). Industry players like Cytokinetics Inc and MyoKardia Inc pioneered direct targeting of the sarcomeric muscle myosins. The first clinical candidate identified for direct targeting of cardiac myosin was Cytokinetics’ omecamtiv mecarbil (OM) (Malik et al., 2011), a cardiac sarcomere activator that currently completed phase III trials to treat systolic heart failure. MyoKardia’s mavacamten (previously MYK-461) is a small-molecule inhibitor of β-cardiac myosin (Green et al., 2016) and is in phase III trials to treat obstructive HCM. This section explores how these small molecules targeted toward the sarcomere can change the DRX↔SRX state equilibrium and impact the energetics and contractility of the muscle.

#### Omecamtiv mecarbil

Although OM was the first therapeutic agent known to target cardiac myosin and activate muscle contractility (Malik et al., 2011), nothing is known about its effects on the SRX state of myosin. However, OM has been shown to inhibit the basal ATPase activity of cardiac myosin (Malik et al., 2011), which hints that it might also induce the SRX state in this system. The crystal structure of OM-bound bovine cardiac myosin has been published, showing that OM binds to a ‘pre-powerstroke’ pocket of the myosin (Planelles-Herrero et al., 2017). Pre-powerstroke is a primed conformation of the lever arm, in which myosin is poised to bind actin and generate force. Interestingly, in the presence of OM, the myosin lever arm appears to adopt a fully primed position, where the lever arm is possibly tilted more in the pre-powerstroke direction. At the same time, the cardiac apo structure has a lever arm position slightly less primed. In different studies (Anderson et al., 2018; Spudich, 2019), it has been hypothesized that a myosin lever-arm position tilted even more in the direction of the pre-powerstroke may give rise to SRX state-like properties. This position of the lever arm may stabilize the closure of the switch II element of the nucleotide-binding pocket, which may slow down the release of ATP hydrolysis products. Given these arguments and in the absence of any data, we hypothesize that OM binding may give rise to SRX state-like properties in myosin. Only future studies will help us test this hypothesis.

#### Mavacamten

Mavacamten was discovered as an ATPase inhibitor that inhibits phosphate release, specifically from the enzymatic pocket of cardiac myosin (Green et al., 2016). Later, Anderson et al., 2018, who demonstrated a depopulation of the SRX state in minipig cardiac fibers harboring the R403Q mutation and in human cardiac fibers with the R663H mutation, showed a re-population of the SRX state when these mutant fibers were treated with mavacamten. Treatment with mavacamten brought the SRX state levels of the mutant fibers back to WT values of 30–40%. By utilizing X-ray diffraction, the authors demonstrated an increase in helical ordering of the WT and mutant thick filaments with mavacamten. Single-particle negative-staining electron microscopy demonstrated that mavacamten stabilized a compact state of myosin in which the heads were folded back against the tail. In the same study, the authors also demonstrated that enzymatically, mavacamten could induce a partial SRX population in shorter myosin constructs that lack the tail region. Related work by Rohde et al., 2018 also demonstrated mavacamten-mediated graded stabilization of SRX populations in bovine cardiac S1 and HMM. In harmony with these observations, by looking at a dose-response of mavacamten, Gollapudi et al., 2020a observed that its potency to populate the SRX state of myosin is the highest in reconstituted myosin thick filaments and least in S1. In the same study, they show that the efficacy of mavacamten is similar in WT and mutant R403Q ventricular β-cardiac and WT atrial α-cardiac myosin (Gollapudi et al., 2020a). They extended their study by showing that the effect of mavacamten to populate the myosin SRX state was diminished in the presence of ADP, consistent with the activation mechanism detailed above for the ADP-bound myosin heads (see section ‘Muscle energetics and the role of ADP in governing the myosin SRX state’). An alternate explanation could be that the binding of mavacamten to myosin may itself be compromised in the presence of ADP in the nucleotide pocket.

Toepfer et al., 2019 demonstrated that truncating mutations in MyBPC depopulated the SRX state in mouse cardiac samples (see section ‘HCM-causing mutations in MyBPC also dysregulate the myosin SRX state’), and this depopulation was reverted when the fibers were treated with mavacamten. Thus, Anderson et al., 2018 first demonstrated mavacamten-mediated re-population of the SRX state in fibers harboring myosin mutations, and Toepfer et al., 2019 extended these observations in fibers harboring mutations in cMyBPC. Reconfirming the hypothesis, Toepfer et al., 2020 recently demonstrated the depopulation of the SRX state by HCM mutations in myosin (see section ‘HCM-causing and other mutations in myosin dysregulate the myosin SRX state’) can be re-populated by treating the samples with mavacamten. They demonstrated this mavacamten-mediated re-population of the SRX state in both mouse left-ventricular tissue samples and human iPSC-derived cardiomyocytes, harboring the same myosin mutations. Very recent work by Nelson et al., 2020 demonstrated the SRX state in slow skeletal myofibrils. The authors demonstrate that slow skeletal myosin in the C-zone is predominantly in the SRX state as compared to the non-MyBPC containing D- and P-zones. They treated the myofibrils with mavacamten and observed a doubling of myosin in the SRX state in the D- and P-zones. In contrast, the SRX population of C-zone myosin remained relatively the same. Thus, myosin motors across the entire thick filament can enter the SRX state whether or not MyBPC is present. However, in the absence of mavacamten, most of the SRX state is concentrated only within the C-zone of the thick filament, hinting at a pivotal role of MyBPC in holding myosin in the energy-conserving SRX state. These emerging findings suggest that the primary mechanism of action of mavacamten is to promote the SRX state in myosin molecules, which is otherwise destabilized in HCM by mutations in myosin and MyBPC.

#### Blebbistatin

Another tool compound, blebbistatin, is a widely used ATPase inhibitor of myosin-II. Blebbistatin binds inside the 50 kDa cleft of myosin and prevents the cleft from closing (Allingham et al., 2005). This results in a weak actin-binding state of myosin and inhibition of rapid hydrolysis and release of nucleotides from myosin. Wilson et al., 2014 demonstrated that blebbistatin caused inhibition of nucleotide turnover in both fast and slow skeletal muscle fibers. They observed a large component of the signal (~40%) decayed at a prolonged rate with a time constant of ~3600 s. They did not observe a change in the population of molecules in the SRX state, but they observed a net decrease in the ATPase rate of the SRX population. A recent study by Gollapudi et al. demonstrated no impact of blebbistatin or its derivatives like para-nitro or para-amino blebbistatin on the SRX population of bovine cardiac reconstitutedthick filaments (Gollapudi et al., 2020b; Gollapudi et al., 2020c). Analogous to Wilson et al., 2014, they observed no change in the SRX population but a significant reduction in the lifetimes of the SRX state. This hints that blebbistatin is likely unable to perturb the DRX↔SRX state equilibrium, but it chiefly works by further slowing down the ATPase rate of the existing SRX-state molecules. Blebbistatin is known to induce a closed-switch II state of myosin (Kovács et al., 2004; Zhao et al., 2008) and induce the helical ordering of thick filaments (Zhao et al., 2008; Xu et al., 2009). However, the studies by Wilson et al., 2014 and Gollapudi et al., 2020b; Gollapudi et al., 2020c potentially decouple the helical ordering or folding-back of myosin and the SRX state signature. Similar results were also seen in three different studies (Anderson et al., 2018; Gollapudi et al., 2020a; Rohde et al., 2018), wherein they observed residual SRX state-like rates in shorter myosin constructs, which were unable to fold-back. These studies demonstrate that SRX state-like rates can also emanate from a conformational ensemble of myosin structures, which may or may not be folded back (Figure 1). Consistent with this idea, in one study by Ma et al., 2018a, blebbistatin has been proposed to stabilize at least two different relaxed states of the myosin, both of which contribute to the ordering of the thick filament.

#### Piperine

Piperine is the primary alkaloid component of black pepper, isolated from an Indian medicinal plant, *Piper nigrum*. Previous studies have shown that piperine can also mitigate obesity and type 2 diabetes in rodent models (Nogara et al., 2016b; BrahmaNaidu et al., 2014; Choi et al., 2013; Kim et al., 2011). Piperine appears to mitigate fat gain during caloric overload and lowers levels of blood glucose and insulin. Nogara et al., 2016b demonstrated that piperine depopulated the SRX state in fast-twitch skeletal fibers, but not in slow-twitch skeletal or cardiac fibers. It increased the ATPase activity of relaxed skinned fibers by ~66%. Piperine was identified as an SRX-state depopulating agent from a high throughput screen by following the changes in the intensity of a fluorescent probe attached to the regulatory light chain of myosin. Data from this work supports a model that piperine promotes thermogenesis in resting muscle by perturbing the DRX↔SRX state equilibrium. In follow-up work, Tolkatchev et al., 2018 discovered that piperine binds the heavy meromyosin (HMM) fragment of myosin, but not the S1-fragment, which lacks the RLC. In this study, they also made a recombinant myosin construct, which could form a stable coiled-coil and bind RLC, thus mimicking the neck region of myosin. This heterotetrameric complex was able to bind piperine, which induced a decrease in the net compactness of the complex as measured by small-angle X-ray scattering. This suggests that piperine’s ability to destabilize the folded-back state of myosin may arise from binding to this portion of the myosin neck region. The authors estimate that shifting heads out of the SRX state into the DRX state would increase the whole-body metabolic rate in humans by 500 kcal/day. This may provide effective therapy for obesity and type 2 diabetes, which together cost ~$500 billion in the USA. However, piperine may not have the specificity needed for use in humans but might be a promising lead compound for discovering similar novel molecules that disrupt the myosin SRX state, thus activating the myosin molecule. Recently Wilson et al., 2021 showed that administration of piperine depopulated the myosin SRX state in human skeletal myosin obtained from biopsies of lean and obsese subjects, without affecting the human cardiac cells. These studies demonstrate that modulating myosin with small molecules to target the DRX↔SRX state equilibrium may be a novel avenue to treat muscle or metabolic disorders.

## Other biochemical tools to modulate the myosin SRX state

### Electrostatically modulating the myosin SRX state

Biochemists love to develop tools to dissect biological processes and gain an in-depth understanding of a process. Anderson et al., 2018 studied the ionic strength dependence on the SRX state of long-tailed 25-hep HMM myosin, and they observed that the fraction of myosin heads in the SRX state increased systematically as the ionic strength decreased. This observation was not seen in either the short-tailed 2-hep HMM or the sS1 (S1 without the RLC) version of the myosin. A similar observation was also made in a bovine cardiac HMM and S1 study (Rohde et al., 2018). In a separate study, a similar decrease in the SRX population with the increased ionic strength of the buffer was also observed in porcine myofibrils, reconstituted myosin thick filaments, and HMM, but not in S1 (Gollapudi et al., 2020a). These studies suggest that an HMM-like molecule containing two S1 heads and the proximal part of the coiled-coil tail, that can form an IHM state, exibit SRX state-like properties that are sensitive to ionic strength. This is consistent with many charge–charge interactions stabilizing the IHM state, more so in full-length myosins than in HMM, demonstrating that the tail region of myosin is necessary to populate its SRX conformation fully. This is consistent with what we described in the ‘Myosin-subfragment 2: the tail tells a tale about the myosin SRX state*’* section.

### Actin–myosin interaction dysregulates the myosin SRX population

Under the fair assumption that a folded compact IHM-like state gives rise to the functional SRX state of myosin, geometrical constraints in an intact sarcomere would likely not allow these folded myosin heads to interact with actin. Rohde et al., 2018, in a reconstituted biochemical experiment, showed that myosins in the SRX configuration could bind to actin, and actin-binding changes the structure to lose its SRX state. For example, for the bovine cardiac HMM, the amplitude of the SRX population decreased, and the ATP turnover rates of these myosins in the SRX state increased in the presence of actin, suggesting an overall shift of the myosins toward a DRX-like state. Moreover, mavacamten, which otherwise populated ~90% of the HMM in the SRX state (as described in the ‘Mavacamten’ section), could only do so to ~50% in the presence of actin. These data potentially suggest that in an in vitro system, when mavacamten-bound myosin is added to a reaction containing a high amount of actin, myosin heads can potentially interact with actin and depopulate the SRX population. An alternate explanation of these observations could be that the actin association of myosin can itself lead to an unbinding of mavacamten from the myosin pocket. As argued above, given the physical constraints of actin and myosin in an actual sarcomere, it remains an open question if, in vivo, folded-back myosin heads can interact with actin.

## Authors’ perspective

### The energy-sparing myosin SRX state may not always arise from the folded-back IHM state

Evidence collected over the last many years has suggested that class-II myosins, both muscle and non-muscle types, can self-sequester into a folded-back state as a mechanism to regulate its function (reviewed in Trivedi et al., 2018 and Alamo et al., 2018). Many low-resolution electron microscopy structures of skeletal-, cardiac-, smooth-, and non-muscle myosins have shown that in this folded-back state, one of the myosin heads of a single myosin dimer, known as the ‘blocked head,’ has its actin-binding domain sequestered into the folded molecule, while the other head is ‘free’ (reviewed in Trivedi et al., 2018 and Alamo et al., 2018). Additionally, from these structures, it has been hypothesized that the ‘blocked head’ interacts with the converter domain of the ‘free head’ and the myosin subfragment 2 to give rise to what is known as the IHM state (reviewed in Trivedi et al., 2018 and Alamo et al., 2018). Very recently, using cryo-electron microscopy, two subsequent seminal works have determined the folded-back IHM structure of smooth muscle myosin at ~4–8 Å resolution (Scarff et al., 2020; Yang et al., 2020). As these fascinating structural discoveries were being made, parallel biochemical investigations discovered that myosin heads in relaxed thick filaments can exist in a SRX state, where it minimizes the use of ATP, thereby providing an energy-saving mechanism (Figure 1). In conjunction with the structural results, these biochemical discoveries led to a proposition, within the central protein structure–function dogma, that the structurally closed IHM state of myosin may give rise to the SRX state and that basically, the SRX state and the IHM state are synonymous. However, this notion has been questioned recently. As discussed earlier, it has been shown in multiple studies that the smaller sub-fragments of cardiac myosins, such as S1 and 2-hep HMM, that are incompetent to achieve the folded-back IHM structure, still exhibit SRX state properties (Anderson et al., 2018; Gollapudi et al., 2020a; Rohde et al., 2018). Moreover, although with less potency than in full-length myosins, mavacamten has been shown to populate the SRX state in these myosin subfragments . With this knowledge combined with homology models of the IHM state, it has been argued that the SRX state of myosin is a functional outcome of a different conformation of the myosin lever arm (Anderson et al., 2018; Spudich, 2019). More precisely, it has been reasoned that a conformation in which the lever arm is tilted more in the pre-stroke direction could give rise to a low-energy consuming state that may resemble the SRX state (Anderson et al., 2018; Spudich, 2019). Together, these pieces of evidence help us postulate that the formation of a proper IHM-like state may not necessarily be required to give rise to the myosin SRX state. In contrast, a classic IHM state, where the myosin lever arm is suggested to be more tilted in the pre-stroke direction, will always give rise to the SRX state. In such a scenario, the additional myosin sub-domain interactions in the IHM state further stabilize the SRX state-like properties. These two possible scenarios that can give SRX state-like behavior have been depicted as schematic structures in Figure 1a.

Similarly, as discussed, the OM-bound crystal structure of cardiac myosin (Planelles-Herrero et al., 2017) also suggests that the myosin lever arm may adopt a more primed position (more tilted in the pre-stroke direction) than the cardiac apo structure. This would predict that OM would promote more myosins in the SRX state, as discussed above. Indeed, OM reduces the basal ATPase of cardiac myosin S1 to the SRX state levels of ATPase activity (Malik et al., 2011). However, two different structural studies, one using fluorescence polarization spectroscopy by Kampourakis et al., 2018 and the other using X-ray fiber diffraction studies of intact rat cardiac fibers by Caremani et al., 2020, have suggested that OM decreases the thick-filament order and helical symmetry and stabilizes the structural ‘on’ state in which myosin heads are more perpendicular to the axis of the thick filament. These results suggest that OM may negatively impact IHM state formation and further challenge the dogma that the formation of the SRX state depends on forming an ordered IHM-like ‘off’ state. Likewise, it is known from multiple structural studies that blebbistatin promotes an IHM-like compact folded-back structure and helical ordering in both isolated purified myosins and skeletal muscle fibers (Zhao et al., 2008; Xu et al., 2009). However, as discussed, blebbistatin or its derivatives like para-nitro or para-amino blebbistatin have no impact on the SRX population of myosin (Gollapudi et al., 2020b), suggesting that there is possibly an uncoupling between the folded back IHM and SRX states. Supportive of this notion, recent small-angle X-ray scattering data suggests that blebbistatin locks myosin into a single conformation between the classic open and the folded IHM states (Gollapudi et al., 2020c).

Altogether, this body of growing evidence directs our thoughts toward an understanding that the SRX state of myosin is probably related to the lever-arm conformation, which gives rise to the super-low usage of ATP by myosin. However, not all perturbations that inhibit myosin’s ATPase activity (e.g., unfolding of myosin itself) fall under the definition of the SRX state. On the other hand, the IHM state is a well-defined state where the myosin heads asymmetrically interact with each other and with the S2-subfragment of myosin. Any folded-back IHM-like (e.g., mavacamten-induced) or open non-IHM-like (e.g., OM-, mavacamten-treated myosin S1) state that has the myosin lever-arm orientation more primed than the pre-stroke condition would lead to the SRX state. While all these seem to be interesting predictions, what is needed to answer these questions would be an atomic-level structure of the folded-back IHM state. To date, there are no high-resolution structures of two-headed myosin HMM in mavacamten, blebbistatin, or OM-bound states. Determining these structures and the lever-arm conformation would resolve the issues discussed above. While it may not be trivial to obtain a high-resolution structure of the IHM state, either by X-ray crystallography or high-resolution cryo-electron microscopy, this must be a high priority goal going forward. Very recently, using cryo-electron microscopy, the folded back IHM structure of smooth muscle myosin at ~4–8 Å resolution was independently determined in two studies, demonstrating that the ‘blocked head’ appears to have its lever arm primed more in the pre-stroke conformation as compared to the ‘free head’. It would be interesting to see whether these structural features are retained in the IHM state of the striated muscle myosins. Meanwhile, one can obtain more information related to our predictions by taking advantage of simultaneous single-turn over kinetics measurements and FRET (Förster resonance energy transfer) distance measurements to measure the SRX state and lever-arm conformations in real time, under the same conditions with purified myosin systems. Recently Pandzic et al., 2020 discussed the use of TIRF (Total Internal Reflection Fluorescence) microscopy as a possibility for measuring popualtion of myosins in the SRX state in human cardiomyocytes. In the absence of high-resolution structures, these biochemical/biophysical measurements would help explore any relationship between the myosin SRX state and the lever-arm conformation. These FRET and TIRF studies could also be extended to more complicated fiber systems by exchanging fluorescent myosins and concurrently measuring the myosin SRX population by already established protocols. Similarly, fiber X-ray diffraction work (Brunello et al., 2020; Caremani et al., 2019; Ma et al., 2018b) in both intact and skinned muscle fibers, which has shed light on the regulatory ‘on’ and ‘off’ states of myosin, if coupled with simultaneous SRX state measurements would be yet another advanced tool to answer ‘how tightly is the myosin SRX state coupled with the structural ‘off’ IHM-like state’?

### Conclusion

**Figure 2.**
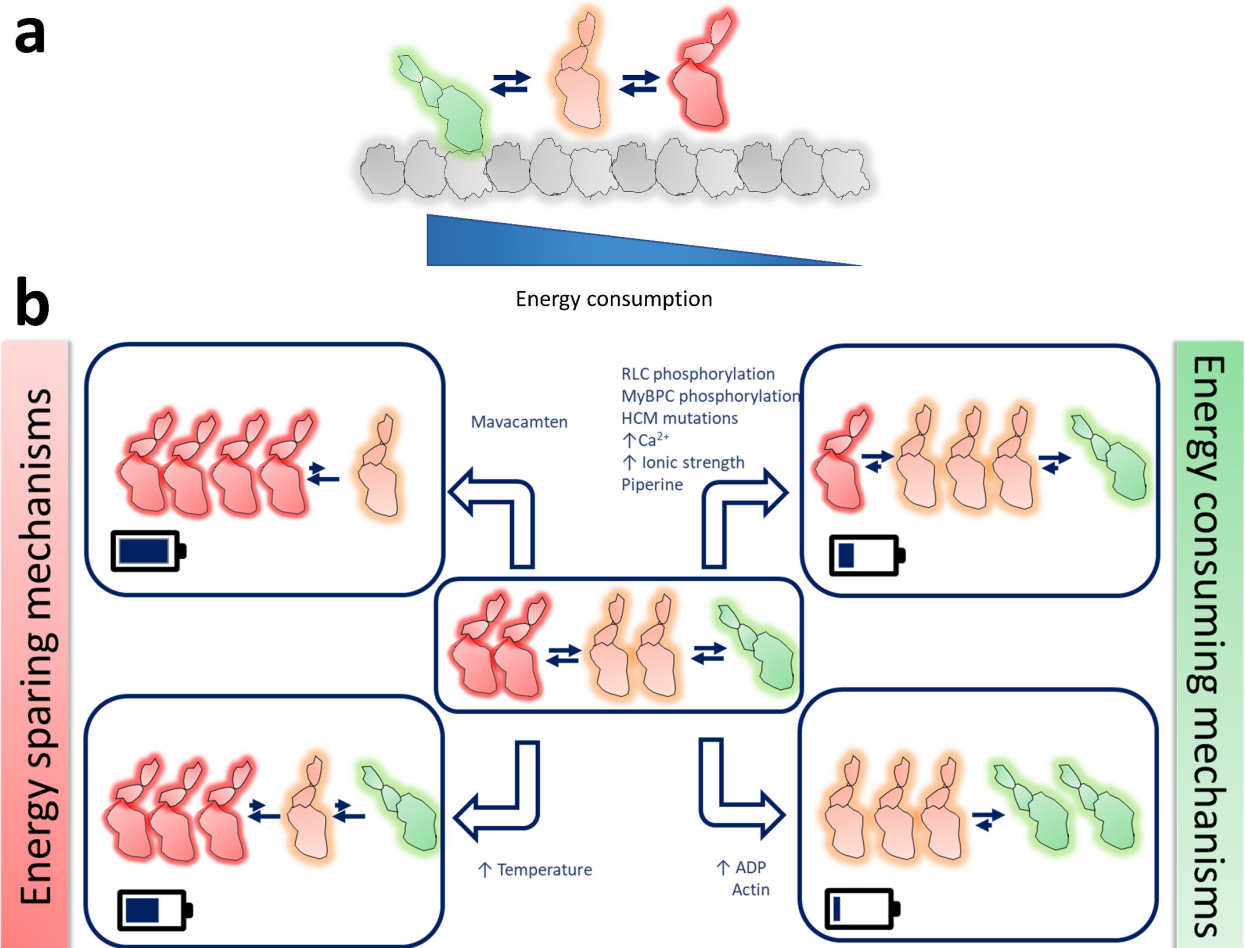
Schematic representation of the possible different functional states of myosin. (**a**) Different states of myosins are arranged in the order of their energy utilization- actin-bound myosin in green, DRX myosin in orange, and SRX myosin in red. (**b**) Graphical representation of how different physiological, pathophysiological, and non-physiological perturbations, as listed, alter the myosin population in these three different states. The small blue arrows next to the different perturbations denote an increase of those parameters. The battery symbol in each box qualitatively resembles the energy saved by the system in each scenario.

A decade of research on the SRX state of myosin has led to some compelling developments in muscle research. It is becoming increasingly clear that the SRX state of myosin plays a pivotal role in the contractility and energetics of the muscle. As we outline in this review, several physiological, pathophysiological, and biochemical factors can modulate the DRX↔SRX state equilibrium (Figure 2), thus fine-tuning the properties of the muscle. As more data is accumulated on this state, it is also becoming clear that the DRX↔SRX state equilibrium is an important target for therapeutic interventions to modulate muscle contractility and metabolism. Other than striated muscle myosins, it is unclear if non-striated muscle and non-muscle myosins exist in a similar DRX↔SRX state equilibrium, although many of these myosins have been shown to exist in an IHM-like state (Alamo et al., 2018; Lee et al., 2018). If so, one can also imagine tweaking the DRX↔SRX state equilibrium for treating other human myosin-related diseases. However, much needs to be learned about the structural state of myosin that leads to the SRX and DRX state biochemical signatures. Structural characterization of the SRX state is undoubtedly an exciting open area of investigation. Finally, it is only after more than a century of muscle research, and through the recent discovery of the SRX state of myosin, we are unraveling the mystery of muscle regulation in healthy and diseased conditions.

## References

[bib1] Abraham TP, Jones M, Kazmierczak K, Liang H-Y, Pinheiro AC, Wagg CS, Lopaschuk GD, Szczesna-Cordary D (2009). Diastolic dysfunction in familial hypertrophic cardiomyopathy transgenic model mice. Cardiovascular Research.

[bib2] Adhikari AST, Sarkar SS, Song D, Kooiker KB, Bernstein D, Spudich JA, Ruppel KM (2019). Hypertrophic cardiomyopathy mutations at the folded-back sequestered β-cardiac myosin S1-S2 and S1-S1 interfaces release sequestered heads and increase myosin enzymatic activity. bioRxiv.

[bib3] Alamo L, Qi D, Wriggers W, Pinto A, Zhu J, Bilbao A, Gillilan RE, Hu S, Padrón R (2016). Conserved intramolecular interactions maintain myosin Interacting-Heads motifs explaining tarantula muscle Super-Relaxed state structural basis. Journal of Molecular Biology.

[bib4] Alamo L, Ware JS, Pinto A, Gillilan RE, Seidman JG, Seidman CE, Padrón R (2017). Effects of myosin variants on interacting-heads motif explain distinct hypertrophic and dilated cardiomyopathy phenotypes. eLife.

[bib5] Alamo L, Pinto A, Sulbarán G, Mavárez J, Padrón R (2018). Lessons from a tarantula: new insights into myosin interacting-heads motif evolution and its implications on disease. Biophysical Reviews.

[bib6] Allingham JS, Smith R, Rayment I (2005). The structural basis of blebbistatin inhibition and specificity for myosin II. Nature Structural & Molecular Biology.

[bib7] Anderson RL, Trivedi DV, Sarkar SS, Henze M, Ma W, Gong H, Rogers CS, Gorham JM, Wong FL, Morck MM, Seidman JG, Ruppel KM, Irving TC, Cooke R, Green EM, Spudich JA (2018). Deciphering the super relaxed state of human β-cardiac myosin and the mode of action of mavacamten from myosin molecules to muscle fibers. PNAS.

[bib8] Barclay CJ (2015). Energetics of contraction. Comprehensive Physiology.

[bib9] BrahmaNaidu P, Nemani H, Meriga B, Mehar SK, Potana S, Ramgopalrao S (2014). Mitigating efficacy of piperine in the physiological derangements of high fat diet induced obesity in Sprague dawley rats. Chemico-Biological Interactions.

[bib10] Brunello E, Fusi L, Ghisleni A, Park-Holohan S-J, Ovejero JG, Narayanan T, Irving M (2020). Myosin filament-based regulation of the dynamics of contraction in heart muscle. PNAS.

[bib11] Burgess SA, Yu S, Walker ML, Hawkins RJ, Chalovich JM, Knight PJ (2007). Structures of smooth muscle myosin and heavy meromyosin in the folded, shutdown state. Journal of Molecular Biology.

[bib12] Caremani M, Brunello E, Linari M, Fusi L, Irving TC, Gore D, Piazzesi G, Irving M, Lombardi V, Reconditi M (2019). Low temperature traps myosin motors of mammalian muscle in a refractory state that prevents activation. Journal of General Physiology.

[bib13] Caremani M, Governali S, Reconditi M, Pinzauti F, Narayanan T, Stienen GJ, Linari M, Lombardi V, Piazzesi G (2020). Modumodulation by inotropic interventions of the regulatory state of the cardiac thick filament in diastole. Biophysical Journal.

[bib14] Chantler PD, Parsons G. J, Shumway S. E (2006). Scallop adductor muscles: Structure and function. Developments in Aquaculture and Fisheries Science.

[bib15] Choi S, Choi Y, Choi Y, Kim S, Jang J, Park T (2013). Piperine reverses high fat diet-induced hepatic steatosis and insulin resistance in mice. Food Chemistry.

[bib16] Colson BA, Petersen KJ, Collins BC, Lowe DA, Thomas DD (2015). The myosin super-relaxed state is disrupted by estradiol deficiency. Biochemical and Biophysical Research Communications.

[bib17] Cooke R (2011). The role of the myosin ATPase activity in adaptive thermogenesis by skeletal muscle. Biophysical Reviews.

[bib18] Craig R, Smith R, Kendrick-Jones J (1983). Light-chain phosphorylation controls the conformation of vertebrate non-muscle and smooth muscle myosin molecules. Nature.

[bib19] Craig R, Woodhead JL (2006). Structure and function of myosin filaments. Current Opinion in Structural Biology.

[bib20] Ebashi S, Endo M (1968). Calcium ion and muscle contraction. Progress in Biophysics and Molecular Biology.

[bib21] Fromherz S, Szent-Györgyi AG (1995). Role of essential light chain EF hand domains in calcium binding and regulation of scallop myosin. PNAS.

[bib22] Fusi L, Huang Z, Irving M (2015). The conformation of myosin heads in relaxed skeletal muscle: implications for Myosin-Based regulation. Biophysical Journal.

[bib23] Gollapudi SK, Yu M, Gan QF, Nag S (2020a). Synthetic thick filaments: a new avenue for better understanding the myosin super-relaxed state in healthy, disease, and mavacamten-treated cardiac systems. The Journal of Biological Chemistry.

[bib24] Gollapudi SK, Sa N, Nag S (2020b). Decoupling the interacting head motif and the super relaxed state of myosin in reconstituted cardiac bipolar thick filaments. Biophysical Journal.

[bib25] Gollapudi SK, Srinivas Chakravarthy WM, Combs AC, Sa N, Langer S, Irving TC, Nag S (2020c). Two classes of myosin inhibitors, blebbistatin and mavacamten, stabilize β-cardiac myosin in different structural and functional states. bioRxiv.

[bib26] Gordon AM, Homsher E, Regnier M (2000). Regulation of contraction in striated muscle. Physiological Reviews.

[bib27] Green EM, Wakimoto H, Anderson RL, Evanchik MJ, Gorham JM, Harrison BC, Henze M, Kawas R, Oslob JD, Rodriguez HM, Song Y, Wan W, Leinwand LA, Spudich JA, McDowell RS, Seidman JG, Seidman CE (2016). A small-molecule inhibitor of sarcomere contractility suppresses hypertrophic cardiomyopathy in mice. Science.

[bib28] Gruen M, Gautel M (1999). Mutations in beta-myosin S2 that cause familial hypertrophic cardiomyopathy (FHC) abolish the interaction with the regulatory domain of myosin-binding protein-C. Journal of Molecular Biology.

[bib29] Guhathakurta P, Prochniewicz E, Thomas DD (2015). Amplitude of the actomyosin power stroke depends strongly on the isoform of the myosin essential light chain. PNAS.

[bib30] Hartman MA, Spudich JA (2012). The myosin superfamily at a glance. Journal of Cell Science.

[bib31] Ho CY, Charron P, Richard P, Girolami F, Van Spaendonck-Zwarts KY, Pinto Y (2015). Genetic advances in Sarcomeric cardiomyopathies: state of the art. Cardiovascular Research.

[bib32] Homburger JR, Green EM, Caleshu C, Sunitha MS, Taylor RE, Ruppel KM, Metpally RP, Colan SD, Michels M, Day SM, Olivotto I, Bustamante CD, Dewey FE, Ho CY, Spudich JA, Ashley EA (2016). Multidimensional structure-function relationships in human β-cardiac myosin from population-scale genetic variation. PNAS.

[bib33] Hooijman P, Stewart MA, Cooke R (2011). A new state of cardiac myosin with very slow ATP turnover: a potential cardioprotective mechanism in the heart. Biophysical Journal.

[bib34] Huang J, Shelton JM, Richardson JA, Kamm KE, Stull JT (2008). Myosin regulatory light chain phosphorylation attenuates cardiac hypertrophy. Journal of Biological Chemistry.

[bib35] Ingwall JS, Weiss RG (2004). Is the failing heart energy starved? on using chemical energy to support cardiac function. Circulation Research.

[bib36] Irving M (2017). Regulation of contraction by the thick filaments in Skeletal Muscle. Biophysical Journal.

[bib37] Jung HS, Burgess SA, Billington N, Colegrave M, Patel H, Chalovich JM, Chantler PD, Knight PJ (2008a). Conservation of the regulated structure of folded myosin 2 in species separated by at least 600 million years of independent evolution. PNAS.

[bib38] Jung HS, Komatsu S, Ikebe M, Craig R (2008b). Head-head and head-tail interaction: a general mechanism for switching off myosin II activity in cells. Molecular Biology of the Cell.

[bib39] Kampourakis T, Zhang X, Sun YB, Irving M (2018). Omecamtiv mercabil and blebbistatin modulate cardiac contractility by perturbing the regulatory state of the myosin filament. The Journal of Physiology.

[bib40] Kazmierczak K, Xu Y, Jones M, Guzman G, Hernandez OM, Kerrick WG, Szczesna-Cordary D (2009). The role of the N-terminus of the myosin essential light chain in cardiac muscle contraction. Journal of Molecular Biology.

[bib41] Kim KJ, Lee MS, Jo K, Hwang JK (2011). Piperidine alkaloids from *Piper retrofractum* Vahl. protect against high-fat diet-induced obesity by regulating lipid metabolism and activating AMP-activated protein kinase. Biochemical and Biophysical Research Communications.

[bib42] Kobayashi T, Solaro RJ (2005). Calcium, thin filaments, and the integrative biology of cardiac contractility. Annual Review of Physiology.

[bib43] Kovács M, Tóth J, Hetényi C, Málnási-Csizmadia A, Sellers JR (2004). Mechanism of blebbistatin inhibition of myosin II. Journal of Biological Chemistry.

[bib44] Lee KH, Sulbarán G, Yang S, Mun JY, Alamo L, Pinto A, Sato O, Ikebe M, Liu X, Korn ED, Sarsoza F, Bernstein SI, Padrón R, Craig R (2018). Interacting-heads motif has been conserved as a mechanism of myosin II inhibition since before the origin of animals. PNAS.

[bib45] Levine RJ, Kensler RW, Yang Z, Stull JT, Sweeney HL (1996). Myosin light chain phosphorylation affects the structure of rabbit skeletal muscle thick filaments. Biophysical Journal.

[bib46] Liu J, Taylor DW, Krementsova EB, Trybus KM, Taylor KA (2006). Three-dimensional structure of the myosin V inhibited state by cryoelectron tomography. Nature.

[bib47] Ma W, Gong H, Irving T (2018a). Myosin head configurations in resting and contracting murine skeletal muscle. International Journal of Molecular Sciences.

[bib48] Ma W, Gong H, Kiss B, Lee EJ, Granzier H, Irving T (2018b). Thick-Filament extensibility in intact skeletal Muscle. Biophysical Journal.

[bib49] Malik FI, Hartman JJ, Elias KA, Morgan BP, Rodriguez H, Brejc K, Anderson RL, Sueoka SH, Lee KH, Finer JT, Sakowicz R, Baliga R, Cox DR, Garard M, Godinez G, Kawas R, Kraynack E, Lenzi D, Lu PP, Muci A, Niu C, Qian X, Pierce DW, Pokrovskii M, Suehiro I, Sylvester S, Tochimoto T, Valdez C, Wang W, Katori T, Kass DA, Shen YT, Vatner SF, Morgans DJ (2011). Cardiac myosin activation: a potential therapeutic approach for systolic heart failure. Science.

[bib50] Manstein DJ, Preller M (2020). Small molecule effectors of myosin function. Advances in Experimental Medicine and Biology.

[bib51] McNamara JW, Li A, Dos Remedios CG, Cooke R (2015). The role of super-relaxed myosin in skeletal and cardiac muscle. Biophysical Reviews.

[bib52] McNamara JW, Li A, Smith NJ, Lal S, Graham RM, Kooiker KB, van Dijk SJ, Remedios CGD, Harris SP, Cooke R (2016). Ablation of cardiac myosin binding protein-C disrupts the super-relaxed state of myosin in murine cardiomyocytes. Journal of Molecular and Cellular Cardiology.

[bib53] McNamara JW, Li A, Lal S, Bos JM, Harris SP, van der Velden J, Ackerman MJ, Cooke R, Dos Remedios CG (2017). MYBPC3 mutations are associated with a reduced super-relaxed state in patients with hypertrophic cardiomyopathy. PLOS ONE.

[bib54] McNamara JW, Singh RR, Sadayappan S (2019). Cardiac myosin binding protein-C phosphorylation regulates the super-relaxed state of myosin. PNAS.

[bib55] Metzger JM, Greaser ML, Moss RL (1989). Variations in cross-bridge attachment rate and tension with phosphorylation of myosin in mammalian skinned skeletal muscle fibers. Implications for twitch potentiation in intact muscle. Journal of General Physiology.

[bib56] Metzger JM, Moss RL (1990). Calcium-sensitive cross-bridge transitions in mammalian fast and slow skeletal muscle fibers. Science.

[bib57] Metzger JM, Moss RL (1991). Kinetics of a ca(2+)-sensitive cross-bridge state transition in skeletal muscle fibers. Effects due to variations in thin filament activation by extraction of troponin C. Journal of General Physiology.

[bib58] Metzger JM, Moss RL (1992). Myosin light chain 2 modulates calcium-sensitive cross-bridge transitions in vertebrate skeletal muscle. Biophysical Journal.

[bib59] Muthu P, Wang L, Yuan CC, Kazmierczak K, Huang W, Hernandez OM, Kawai M, Irving TC, Szczesna-Cordary D (2011). Structural and functional aspects of the myosin essential light chain in cardiac muscle contraction. The FASEB Journal.

[bib60] Naber N, Cooke R, Pate E (2011). Slow myosin ATP turnover in the super-relaxed state in tarantula muscle. Journal of Molecular Biology.

[bib61] Nag S, Trivedi DV, Sarkar SS, Adhikari AS, Sunitha MS, Sutton S, Ruppel KM, Spudich JA (2017). The myosin Mesa and the basis of hypercontractility caused by hypertrophic cardiomyopathy mutations. Nature Structural & Molecular Biology.

[bib62] Nelson SR, Li A, Beck-Previs S, Kennedy GG, Warshaw DM (2020). Imaging ATP consumption in resting skeletal muscle: one molecule at a time. Biophysical Journal.

[bib63] Nogara L, Naber N, Pate E, Canton M, Reggiani C, Cooke R (2016a). Spectroscopic studies of the super relaxed state of skeletal muscle. PLOS ONE.

[bib64] Nogara L, Naber N, Pate E, Canton M, Reggiani C, Cooke R (2016b). Piperine's mitigation of obesity and diabetes can be explained by its up-regulation of the metabolic rate of resting muscle. PNAS.

[bib65] Onishi H, Wakabayashi T (1982). Electron microscopic studies of myosin molecules from chicken gizzard muscle I: the formation of the intramolecular loop in the myosin tail. The Journal of Biochemistry.

[bib66] Pandzic E, Morkel CA, Li A, Cooke R, Whan RM, Dos Remedios CG (2020). Nanomolar ATP binding to single myosin cross-bridges in rigor: a molecular approach to studying myosin ATP kinetics using single human cardiomyocytes. Biophysical Reviews.

[bib67] Phung LA, Karvinen SM, Colson BA, Thomas DD, Lowe DA (2018). Age affects myosin relaxation states in skeletal muscle fibers of female but not male mice. PLOS ONE.

[bib68] Phung LA, Foster AD, Miller MS, Lowe DA, Thomas DD (2020). Super-relaxed state of myosin in human skeletal muscle is fiber-type dependent. American Journal of Physiology-Cell Physiology.

[bib69] Planelles-Herrero VJ, Hartman JJ, Robert-Paganin J, Malik FI, Houdusse A (2017). Mechanistic and structural basis for activation of cardiac myosin force production by omecamtiv mecarbil. Nature Communications.

[bib70] Rohde JA, Roopnarine O, Thomas DD, Muretta JM (2018). Mavacamten stabilizes an autoinhibited state of two-headed cardiac myosin. PNAS.

[bib71] Sa N, Tomasic I, Gollapudi S, Nag S (2019). Abstract 340: myosin regulatory light chain: a major player in defining the ‘OFF’ State of Cardiac Myosin. Circulation Research.

[bib72] Sarkar SS, Trivedi DV, Morck MM, Adhikari AS, Pasha SN, Ruppel KM, Spudich JA (2020). The hypertrophic cardiomyopathy mutations R403Q and R663H increase the number of myosin heads available to interact with actin. Science Advances.

[bib73] Scarff CA, Carrington G, Casas-Mao D, Chalovich JM, Knight PJ, Ranson NA, Peckham M (2020). Structure of the shutdown state of myosin-2. Nature.

[bib74] Sitbon YH, Kazmierczak K, Liang J, Yadav S, Veerasammy M, Kanashiro-Takeuchi RM, Szczesna-Cordary D (2020). Ablation of the N terminus of cardiac essential light chain promotes the super-relaxed state of myosin and counteracts hypercontractility in hypertrophic cardiomyopathy mutant mice. The FEBS Journal.

[bib75] Spudich JA (2019). Three perspectives on the molecular basis of hypercontractility caused by hypertrophic cardiomyopathy mutations. Pflügers Archiv - European Journal of Physiology.

[bib76] Stewart MA, Franks-Skiba K, Chen S, Cooke R (2010). Myosin ATP turnover rate is a mechanism involved in thermogenesis in resting skeletal muscle fibers. PNAS.

[bib77] Szent-Györgyi AG (1975). Calcium regulation of muscle contraction. Biophysical Journal.

[bib78] Toepfer C, Caorsi V, Kampourakis T, Sikkel MB, West TG, Leung M-C, Al-Saud SA, MacLeod KT, Lyon AR, Marston SB, Sellers JR, Ferenczi MA (2013). Myosin regulatory light chain (RLC) Phosphorylation change as a modulator of cardiac muscle contraction in disease. Journal of Biological Chemistry.

[bib79] Toepfer CN, Wakimoto H, Garfinkel AC, McDonough B, Liao D, Jiang J, Tai AC, Gorham JM, Lunde IG, Lun M, Lynch TL, McNamara JW, Sadayappan S, Redwood CS, Watkins HC, Seidman JG, Seidman CE (2019). Hypertrophic cardiomyopathy mutations in *MYBPC3* dysregulate myosin. Science Translational Medicine.

[bib80] Toepfer CN, Garfinkel AC, Venturini G, Wakimoto H, Repetti G, Alamo L, Sharma A, Agarwal R, Ewoldt JF, Cloonan P, Letendre J, Lun M, Olivotto I, Colan S, Ashley E, Jacoby D, Michels M, Redwood CS, Watkins HC, Day SM, Staples JF, Padrón R, Chopra A, Ho CY, Chen CS, Pereira AC, Seidman JG, Seidman CE (2020). Myosin sequestration regulates sarcomere function, cardiomyocyte energetics, and metabolism, informing the pathogenesis of hypertrophic cardiomyopathy. Circulation.

[bib81] Tolkatchev D, Elnatan D, Nogara L, Ly T, Naber N, Haak K, Meech R, Cooke R, Kostyukova AS (2018). Piperine, an alkaloid inhibiting the super-relaxed state of myosin, binds to the myosin regulatory light chain. Archives of Biochemistry and Biophysics.

[bib82] Trivedi DV, Adhikari AS, Sarkar SS, Ruppel KM, Spudich JA (2018). Hypertrophic cardiomyopathy and the myosin mesa: viewing an old disease in a new light. Biophysical Reviews.

[bib83] Trybus KM, Huiatt TW, Lowey S (1982). A bent monomeric conformation of myosin from smooth muscle. PNAS.

[bib84] Trybus KM (1994). Role of myosin light chains. Journal of Muscle Research and Cell Motility.

[bib85] van der Velden J, Papp Z, Boontje NM, Zaremba R, de Jong JW, Janssen PM, Hasenfuss G, Stienen GJ (2003). Myosin light chain composition in non-failing donor and end-stage failing human ventricular myocardium. Advances in Experimental Medicine and Biology.

[bib86] Wendt T, Taylor D, Messier T, Trybus KM, Taylor KA (1999). Visualization of Head–Head Interactions in the Inhibited State of Smooth Muscle Myosin. Journal of Cell Biology.

[bib87] Wendt T, Taylor D, Trybus KM, Taylor K (2001). Three-dimensional image reconstruction of dephosphorylated smooth muscle heavy meromyosin reveals asymmetry in the interaction between myosin heads and placement of subfragment 2. PNAS.

[bib88] Wilson C, Naber N, Pate E, Cooke R (2014). The myosin inhibitor blebbistatin stabilizes the super-relaxed state in skeletal muscle. Biophysical Journal.

[bib89] Wilson C, Naber N, Cooke R (2021). The role of the super-relaxed state of myosin in human metabolism. Metabolism Open.

[bib90] Woodhead JL, Zhao FQ, Craig R, Egelman EH, Alamo L, Padrón R (2005). Atomic model of a myosin filament in the relaxed state. Nature.

[bib91] Woodhead JL, Zhao FQ, Craig R (2013). Structural basis of the relaxed state of a Ca2+-regulated myosin filament and its evolutionary implications. PNAS.

[bib92] Xu S, Offer G, Gu J, White HD, Yu LC (2003). Temperature and ligand dependence of conformation and helical order in myosin filaments. Biochemistry.

[bib93] Xu S, White HD, Offer GW, Yu LC (2009). Stabilization of helical order in the thick filaments by blebbistatin: further evidence of coexisting multiple conformations of myosin. Biophysical Journal.

[bib94] Yadav S, Kazmierczak K, Liang J, Sitbon YH, Szczesna-Cordary D (2019). Phosphomimetic-mediated *in vitro* rescue of hypertrophic cardiomyopathy linked to R58Q mutation in myosin regulatory light chain. The FEBS Journal.

[bib95] Yang S, Tiwari P, Lee KH, Sato O, Ikebe M, Padrón R, Craig R (2020). Cryo-EM structure of the inhibited (10S) form of myosin II. Nature.

[bib96] Yu H, Chakravorty S, Song W, Ferenczi MA (2016). Phosphorylation of the regulatory light chain of myosin in striated muscle: methodological perspectives. European Biophysics Journal.

[bib97] Zhao FQ, Padrón R, Craig R (2008). Blebbistatin stabilizes the helical order of myosin filaments by promoting the switch 2 closed state. Biophysical Journal.

[bib98] Zoghbi ME, Woodhead JL, Moss RL, Craig R (2008). Three-dimensional structure of vertebrate cardiac muscle myosin filaments. PNAS.

